# Angiogenesis in myelodysplastic syndromes

**DOI:** 10.1038/sj.bjc.6693515

**Published:** 1999-12

**Authors:** G Pruneri, F Bertolini, D Soligo, N Carboni, A Cortelezzi, P F Ferrucci, R Buffa, G Lambertenghi-Deliliers, F Pezzella

**Affiliations:** Second Division of Pathology, Bone Marrow Transplantation Unit andDiagnostic Hematology, IRCCS Maggiore Hospital; Hematology-Oncology Unit, IRCCS European Institute of Oncology, Milan, Italy; Medical Oncology, IRCCS Maugeri Foundation, Pavia, Italy; Department of Histopathology, University College London, UK

**Keywords:** myelodysplasia, angiogenesis, leukaemia, VEGF

## Abstract

It is now well established that solid tumour growth depends on angiogenesis. However, less is known about the generation of new vessels in haematological malignancies and, in particular, in preleukaemic-myelodysplastic syndromes (MDS). In this study, bone marrow microvessel density (MVD) was assessed by immunohistochemistry and compared in trephine biopsies from 14 controls, five infectious disease (ID), 82 MDS, 15 acute myeloid leukaemia (AML) and 14 myeloproliferative disorder (MPD) patients. Statistical analysis (*P* < 0.001) demonstrated that MDS MVD was higher than in controls and ID (21 ± 9 vs 6 ± 2 and 10 ± 8 respectively) but lower than AML (30 ± 12) and MPD (40 ± 12). Among MDS-FAB subtypes, MVD was significantly higher in RAEB-t, CMML and fibrosis subsets compared to RA, RARS and RAEB subsets (*P* = 0.008). To further investigate angiogenesis machinery, the expression of vascular endothelial growth factor (VEGF) was evaluated by means of immunohistochemistry in control, MDS, AML and MPD biopsies. Even though VEGF mRNA expression was reported in the past in AML cell cultures and cell lines, in our samples VEGF expression was found to be particularly strong in most of the megakaryocytes but significantly less prominent in other cell populations including blasts. Since our findings suggest a correlation between angiogenesis and progression to leukaemia, additional work is now warranted to determine what regulates the generation of new vessels in MDS and leukaemia. © 1999 Cancer Research Campaign


*British Journal of Cancer* (1999) **81**(8), 1398–1401
© 1999 Cancer Research Campaign
Article no. bjoc.1999.0858

### Angiogenesis in myelodysplastic syndromes

**G Pruneri[1]*, F Bertolini[4,5]*, D Soligo[2], N Carboni[1], A Cortelezzi[3], PF Ferrucci[4], R Buffa[1], G Lambertenghi-Deliliers[2] and F Pezzella[6]**

[1]Second Division of Pathology, [2]Bone Marrow Transplantation Unit and [3]Diagnostic Hematology, IRCCS Maggiore Hospital; [4]Hematology-Oncology Unit, IRCCS European Institute of Oncology, Milan, Italy; [5]Medical Oncology, IRCCS Maugeri Foundation, Pavia, Italy; [6]Department of Histopathology, University College London, UK

**Summary** It is now well established that solid tumour growth depends on angiogenesis. However, less is known about the generation of new vessels in haematological malignancies and, in particular, in preleukaemic-myelodysplastic syndromes (MDS). In this study, bone marrow microvessel density (MVD) was assessed by immunohistochemistry and compared in trephine biopsies from 14 controls, five infectious disease (ID), 82 MDS, 15 acute myeloid leukaemia (AML) and 14 myeloproliferative disorder (MPD) patients. Statistical analysis ($P < 0.001$) demonstrated that MDS MVD was higher than in controls and ID (21 ± 9 vs 6 ± 2 and 10 ± 8 respectively) but lower than AML (30 ± 12) and MPD (40 ± 12). Among MDS-FAB subtypes, MVD was significantly higher in RAEB-t, CMML and fibrosis subsets compared to RA, RARS and RAEB subsets ($P = 0.008$). To further investigate angiogenesis machinery, the expression of vascular endothelial growth factor (VEGF) was evaluated by means of immunohistochemistry in control, MDS, AML and MPD biopsies. Even though VEGF mRNA expression was reported in the past in AML cell cultures and cell lines, in our samples VEGF expression was found to be particularly strong in most of the megakaryocytes but significantly less prominent in other cell populations including blasts. Since our findings suggest a correlation between angiogenesis and progression to leukaemia, additional work is now warranted to determine what regulates the generation of new vessels in MDS and leukaemia. © 1999 Cancer Research Campaign

**Keywords**: myelodysplasia; angiogenesis; leukaemia; VEGF

In vitro and in vivo laboratory studies and clinical data have generated ample evidence that neovascularization supports solid tumour viability and growth (Folkman, 1995). Along this line, a recent paper from Perez-Atayde et al (1997) has suggested that angiogenesis might also play a pivotal role in human leukaemia. In fact, these authors have found a median of 42 vessels per 200× field in bone marrow (BM) biopsies from paediatric acute lymphoblastic leukaemia (ALL) patients compared to a median of 6 vessels in normal controls. Differences between relapsed and non-relapsed patients were not significant, and chemotherapy did not decrease the number of vessels per field. Consistent with these data, increased levels of the endothelial cell mitogenic molecule basic fibroblast growth factor (bFGF) have been reported in the urine of paediatric ALL patients compared to controls. Again, chemotherapy did not significantly decrease bFGF concentration in the patients' urine. More indirect evidence of neovascularization in leukaemia comes from the observation that vascular endothelial growth factor (VEGF) is expressed by acute myeloid leukaemia (AML) cells and leukaemic cell lines and possibly act as a paracrine growth factor in the development of myeloid leukaemia (Fiedler et al, 1997). Moreover, it has been proposed that angiogenesis and VEGF could play a cardinal role also in lymphoma (Foss et al, 1997; Salven et al, 1997) and myeloma (Vacca et al, 1994) development. We were interested in determining whether conversion of normal cells into preleukaemic-myelodysplastic (MDS), and ultimately leukaemic cells is a multistep process requiring the generation of new blood vessels. To this aim, we evaluated microvessel density (MVD) and VEGF expression in BM biopsies from healthy controls, infectious disease (ID), MDS, AML and myeloproliferative disorder (MPD) patients.

*Received 3 March 1999*
*Accepted 25 May 1999*

*Correspondence to:* F Bertolini, Hematology-Oncology Unit, European Institute of Oncology, via Ripamonti 435, 20141 Milan, Italy

### MATERIALS AND METHODS

We evaluated 133 paraffin-embedded BM biopsies collected from 1988 to 1998. BM biopsies were from 14 controls (solid cancer or Hodgkin's disease patients undergoing staging or follow-up and found to be free of neoplasia at histological examination), five ID, 85 MDS, 15 AML and 14 MPD patients. Among MDS patients, the median age was 67 years (range 26–86), 34% patients were female and 66% male. MDS, AML or MPD diagnosis was made according to FAB classification by means of BM aspirate stained with May–Grunewald–Giemsa and trephine biopsy stained with haematoxylin–eosin, Gomori and Giemsa. Ninety-four biopsies were fixed in B5, decalcified by EDTA (Mielodec, Bio-Optica, Milan, Italy) and embedded in paraffin in Milan, 39 were fixed in 10% buffered formalin, decalcified in 10% formic acid and embedded in paraffin in London. The immunohistochemical analysis was made by means of the avidin–biotin peroxidase complex (ABC) method using the 3,3′-diaminobenzidine tetrahydrochloride chromogen. The 4-μm-thick sections were immuno–-stained with the following monoclonal antibodies: CD34-reactive QBEnd/10 (1:10 working concentration, Signet Laboratories Inc., Dedham, MA, USA), CD31-reactive 1A10 (1:50 working concentration, Novocastra Laboratories Ltd, Newcastle upon Tyne, UK),

*Contributed equally to this work.

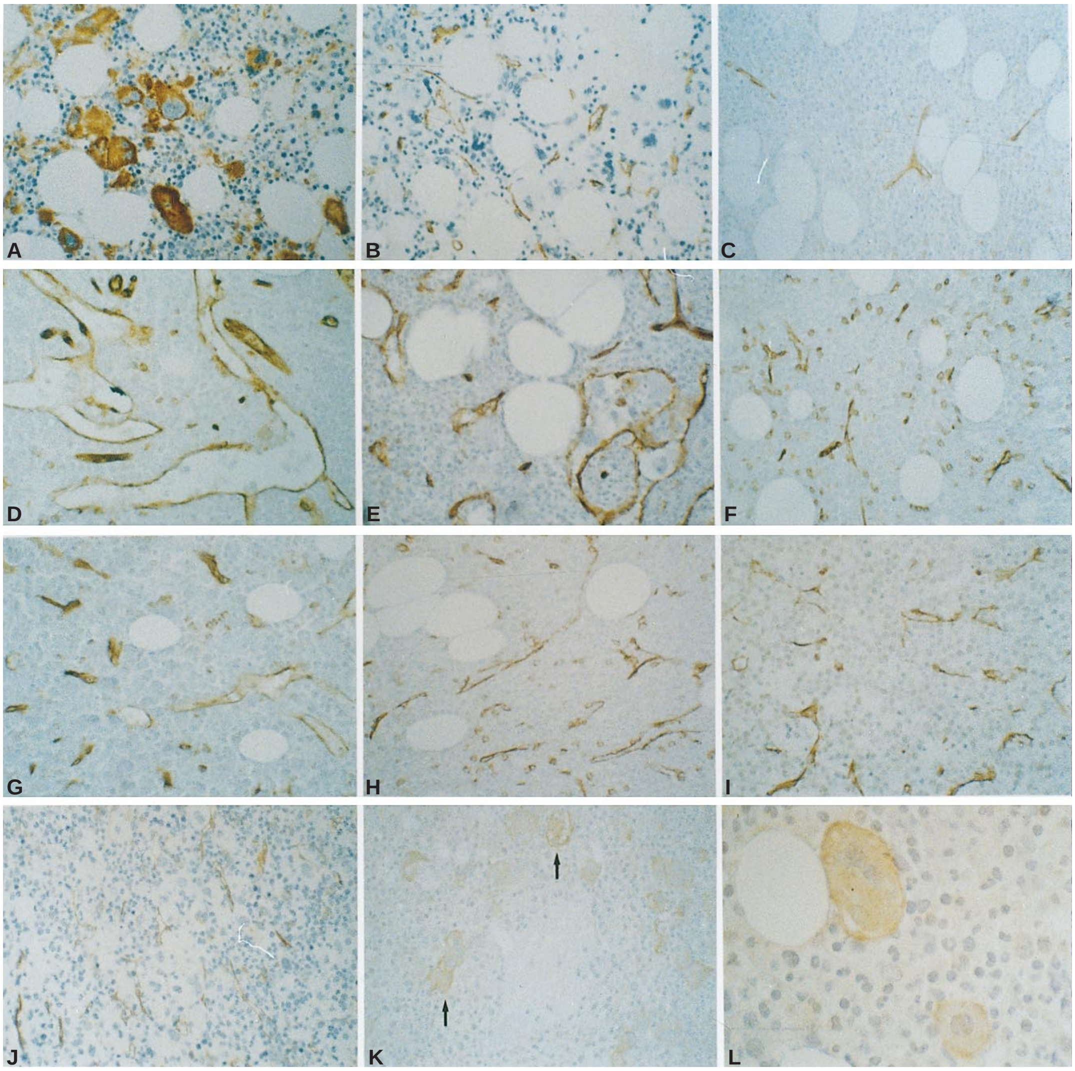

**Figure 1** Examples of immunohistochemical results, 25× original magnification if not otherwise indicated, haematoxylin counterstain. CD31 and CD34 expression in the same field of a MPD BM biopsy. CD31 was expressed in a very large population of BM cells, including megakaryocytes and myeloid cells (**A**). Thus, in most cases, accurate MVD enumeration was not feasible. Conversely, CD34 was expressed only by haematopoietic progenitor and endothelial cells, thus allowing a more accurate MVD evaluation (**B**). MVD in representative BM biopsies from healthy controls (**C**), MDS (**D**–**H**), AML (**I**) and MPD (CML) patients (**J**). VEGF expression in representative BM biopsies from MDS patients. VEGF expression was limited to the cytoplasm of megakaryocytes (some of which indicated by arrows in **K**), with higher intensity along the cell membrane (**L**, 63× original magnification)

VEGF-reactive Ab-3 (1:100 working concentration, Oncogene Research Products, Cambridge, MA, USA). Slides were slightly counterstained with haematoxylin. For antigen retrieval, the slides were placed in a 0.1 M citrate buffer at pH 6.0 (VEGF and CD31), or in a 0.001 M EDTA buffer at pH 8.0 (CD34) and underwent three (VEGF and CD31) or four (CD34) 5-min 780W cycles at 90° in a microwave oven. According to the manufacturer, biopsies from fetal kidneys and hormone-secreting prostate cancer patients were evaluated as VEGF-positive controls. The substitution of the primary antibody with non-immune mouse serum was used as negative control. MVD enumeration was made according to Perez-Atayde et al (1997): in each sample a mean of 12 ± 7 microscopic fields (median 12, range 3–47) was evaluated at 250× magnification, each field representing an area of 0.72 $mm^2$. Any brown staining endothelial cell or endothelial cell cluster that was clearly separated from adjacent microvessels was considered a single, countable microvessel and vessel lumens were not a prerequisite to define a structure as a microvessel. Immunostaining of blasts and arterioles, clearly identifiable by the round shape and the presence of a central nucleus with one or more nucleoli, and by the presence of the media layer, were not counted. For each case, the median value of vessels and the field with the highest number of vessels (‘hot spot’) were recorded. Two readers evaluated slides in a blind fashion.

Statistical comparisons were performed using the linear regression analysis, the *t*-test, analysis of variance (ANOVA) and Student–Neumann–Keuls test in paired studies and the non-parametric analyses of Mann–Whitney, Wilcoxon and Kruskal–Wallis in non-paired studies. Values of *P* lower than 0.05 were considered as statistically significant.

*© 1999 Cancer Research Campaign*

**Table 1** Mean (± 1 s.d.) MVD and hot spots in control, ID, MDS, AML and MPD patients

| | *n* | MVD (per 250× field) | Hot spots |
|---|---|---|---|
| Controls | 14 | 6 ± 2 | 12 ± 4 |
| ID | 5 | 10 ± 8 | 19 ± 13 |
| MDS (overall) | 82 | 21 ± 9[a] | 34 ± 13[a] |
| RA | 14 | 21 ± 7 | 33 ± 9 |
| RARS | 13 | 19 ± 8 | 35 ± 17 |
| RAEB | 35 | 19 ± 8 | 30 ± 10 |
| RAEB-t | 11 | 28 ± 12 | 41 ± 17 |
| CMML | 4 | 28 ± 5 | 39 ± 4 |
| Fibrosis | 4 | 29 ± 8 | 43 ± 14 |
| AML | 15 | 30 ± 12[a,b] | 43 ± 19[a,b] |
| MPD | 14 | 40 ± 14[a,b,c] | 57 ± 19[a,b,c] |

[a]$P < 0.05$ vs controls and ID, [b]$P < 0.05$ vs MDS, [c]$P < 0.05$ vs AML, by analysis of variance and Student–Neumann–Keuls test.

### RESULTS

Best immunohistochemical results were obtained with anti-CD34 monoclonal antibody QBEnd/10, which depicted endothelial cells and a variable number of blasts. With this antibody, MVD intra-reader variability was found to be 14 ± 10% ($r$ = 0.879), thus indicating good reproducibility. By contrast, anti-CD31 invariably immunostained myeloid, lymphoid cells and megakaryocytes besides vessels, thus making difficult the microvessels count (Figure 1 A, B). Therefore, only the results obtained with the QBEnd/10 antibody are given (Figure 1 B–J). In three out of 85 MDS cases, CD34+ BM blast frequency was so high that MVD evaluation by means of CD34+ vessel enumeration was not feasible. Consequently, throughout the study we report data from 82 MDS cases. As shown in Table 1, MVD and hot spots were similar in controls and ID, significantly higher in MDS than in controls and ID, significantly lower in MDS than in AML and MPD ($P < 0.001$ by ANOVA and Student–Neumann–Keuls test). Among FAB-related MDS subsets, MVD was significantly higher in the RAEB-t, CMML and fibrosis subsets compared to RA, RARS and RAEB subsets ($P = 0.008$). No significant differences were found between the RAEB-t, CMML and fibrosis MDS-related subsets and AML patients. A detailed evaluation of the international prognostic scoring system (IPSS; Greenberg et al, 1997) was available in 29 out of 82 evaluated MDS patients, and in this subset MVD did not correlate significantly with age, gender, presence of chromosome abnormality and PLT count, whereas a weak trend indicated a higher MVD in patients with higher blast frequency, white blood cell (WBC) count and lower haemaglobin (Hb) levels ($r$ = 0.20, 0.28 and 0.29 respectively). Detailed data about progression to AML were available for 22 patients. In this small group, MVD differences between patients who developed AML and patients who had stable MDS were not significant.

In normal controls the vessels showed a straight shape (Figure 1C), whereas in MDS, AML and MPD there were basically three morphological types of vessels: large vessels with visible lumina and very irregular, branching shape (Figure 1 D, E), sinusoids-like vessels (Figure 1B), and small vessels without discernible lumina (Figure 1 I, J), which have been defined ‘endothelial sprouts’ by Perez-Atayde et al (1997). Whereas these morphological variants were usually detectable in the same case, the first type predominated in MDS, the second in MPD and the third in AML. VEGF expression was usually found in the cytoplasm of megakaryocytes and histiocytes in normal controls, MDS and MPD, whereas the blast compartment was usually unreactive (Figure 1 K, L). In particular, among AML patients we have observed few scattering VEGF expressing blasts representing less than 1% of the total blast population.

### DISCUSSION

The seminal observation by Perez-Atayde et al (1997) of neovascularization in ALL BM environment prompted us to study MVD in preleukaemia-MDS BM biopsies. In fact, it has been suggested in the past that the growth of primary solid tumours is strictly dependent on their ability to induce angiogenesis from the surrounding vasculature, so that tumour progression, including invasion and metastasis, involves two different phases, prevascular and vascular (Gimbrone et al, 1972; Folkman, 1990). Our finding that MDS has intermediate MVD levels between controls and AML might suggest a correlation between angiogenesis and progression to leukaemia, and it seems unlikely that increased MVD was solely due to hypercellularity. In fact, MVD observed in five cases with reactive hypercellular BM was comparable to that of normocellular control trephines. The correlation between angiogenesis and progression to leukaemia is further supported by the observation that MDS patients with enhanced blasts accumulation and, in particular, the RAEB-t in addition to CMML and fibrosis subsets, show an increased MVD. Regarding the CMML and fibrosis MDS subsets, our finding of an increased MVD compared to RA, RARS and RAEB subsets correlates well with recent observations of VEGF generation by solid tumour-associated macrophages (Lewis et al, 1995) and fibroblasts (Foss et al, 1997; Fukumura et al, 1998). In fact, VEGF is currently considered the most relevant and the only endothelium-specific one among already known endothelial cell mitogenic factors, and it seems to be involved in the vascular phase of many different neoplastic diseases (Ferrara and Davis-Smyth, 1997). Given that a recent report (Fiedler et al, 1997) has demonstrated VEGF mRNA generation from AML BM cells and leukaemic cell lines and VEGF protein generation in AML cell culture, one can speculate that leukaemic or even preleukaemic blasts might be among the principal providers of VEGF in the BM environment. On the other hand, we have evaluated BM biopsies from controls, ID, MDS AML and MPD and have found that VEGF expression is particularly high in megakaryocyes and, on some occasions, in macrophages, but markedly less relevant or otherwise below the detection limit of our procedure in MDS and AML blasts. This finding is in accordance with previous reports (Mohle et al, 1997; Banks et al, 1998) indicating relevant VEGF expression along the megakaryocytic differentiation pathway, and also suggests that MDS or leukaemic blasts might not produce clinically relevant amounts of VEGF. Interestingly, in one particular MDS case a remarkably high frequency of VEGF-positive megakaryocytes was associated with high median MVD (26.5) and hot spot (40). Thus, we are currently evaluating whether megakaryocytes play a crucial role in MDS-related angiogenesis.

In a very recent paper, Fukumura et al (1998) have reported about transgenic mice expressing the green fluorescent protein (GFP) under the control of the promoter for VEGF. In this elegant model, spontaneous tumours induced by oncogene expression show strong stromal, but not tumour, expression of GFP, and the predominant GFP-positive cells are fibroblasts. This finding implies that the VEGF promoter might be activated by the tumour

 © 1999 Cancer Research Campaign microenvironment, and suggests a crucial role for stromal cell collaboration in tumour angiogenesis. In this context, the evaluation of the angiogenic potential of BM stromal cells from MDS, AML and MPD patients remains a future challenge. We are currently evaluating VEGF receptors expression and the generation of other endothelial cell mitogenic factors among different BM cells of MDS, AML and MPD patients to gain more insight into the steps from normal to leukaemic BM microenvironment.

Although the clinical and prognostic relevance of neovascularization in MDS, AML and MPD remains to be fully evaluated, different clinical observations have already indicated that MVD (Weidner et al, 1991; Graham et al, 1994; Vacca et al, 1994; Fernandez-Acernero et al, 1998), bFGF (Nguyen et al, 1994) and/or VEGF (Salven et al, 1997; Linderholm et al, 1998) levels correlate well with tumour staging and outcome in a number of malignancies. In this context, neoangiogenesis in haematological neoplastic diseases is of particular interest, because resting endothelial cells are known to generate stem cell factor (Yamaguchi et al, 1996) and Flt3-ligand (Solanilla et al, 1998) and VEGF-stimulated endothelial cells produce granulocyte–macrophage colony-stimulating factor (GM-CSF; Fiedler et al, 1997). These cytokines, in turn, act as growth factors for haematopoietic progenitors and possibly for neoplastic blasts. In conclusion, our data support an important role of neo angiogenesis in MDS, AML and MPD. Recent data obtained in animal models have demonstrated that anti-angiogenic therapy is highly promising in different experimental neoplastic diseases (Folkman, 1995; Bohem et al, 1997). However, anti-angiogenic therapy may not be able to induce tumour regression but could inhibit further growth by a mechanism of 'tumour stabilization' (Harris, 1997). In this frame of reference, it would be interesting not only to evaluate in experimental leukaemia models the effect of anti-angiogenic drugs, alone or in combination with established chemotherapy regimens, but also whatever anti-angiogenic treatment could have a role in delaying or even preventing disease progression in MDS patients.

### ACKNOWLEDGEMENTS

The authors would like to thank medical and nursing staff at IRCCS Maggiore Hospital, IRCCS European Institute of Oncology and University College London for providing control, ID, MDS, AML and MPD samples. We are also indebted to Patrick Maisonneuve for the statistical analysis, Domenico Delia and Carmelo Carlo-Stella for critical reading of the manuscript and useful suggestions during the study.

### REFERENCES

Banks RE, Forbes MA, Kinsey SE, Stanley A, Ingham E, Walters C and Selby PJ (1998) Release of the angiogenic cytokine vascular endothelial growth factor (VEGF) from platelets: significance for VEGF measurements and cancer biology. *Br J Cancer* **77**: 956–964

Bohem T, Folkman J, Browder T and O'Reilly MS (1997) Antiangiogenic therapy of experimental cancer does not induce acquired drug resistance. *Nature* **390**: 404–407

Fernandez-Acenero MJ, Gonzalez JF, Galindo Gallego M and Aragoncillo Ballestreros P (1998) Vascular enumeration as a significant prognosticator for invasive breast carcinoma. *J Clin Oncol* **16**: 1684–1688

Ferrara N and Davis-Smyth T (1997) The biology of vascular endothelial growth factor. *Endocrine Rev* **18**: 4–25

Fiedler W, Graeven U, Ergun S, Verago S, Kilic N, Stockschlader M and Hossfeld DK (1997) Vascular endothelial growth factor, a possible paracrine factor in human acute myeloid leukemia. *Blood* **89**: 1870–1875

Folkman J (1990) What is the evidence that tumors are angiogenesis dependent? *J Natl Cancer Inst* **82**: 4–6

Folkman J (1995) Angiogenesis in cancer, vascular, rheumatoid and other diseases. *Nature Med* **1**: 27–31

Foss HD, Araujo I, Demel G, Klotzback H, Hummel M and Stein H (1997) Expression of vascular endothelial growth factor in lymphomas and Castleman's disease. *J Pathol* **183**: 44–50

Fukumura D, Xavier R, Sugiura T, Chen Y, Park E, Lu N, Selig M, Nielsen G, Taksir T, Jain RK and Seed B (1998) Tumor induction of VEGF promoter activity in stromal cells. *Cell* **94**: 715–725

Gimbrone MA, Leapman SB, Cotran RS and Folkman J (1972) Tumor dormancy in vivo by prevention of neovascularization. *J Exp Med* **136**: 261–276

Graham C, Rivers J, Kerbel R, Stankiewicz K and White W (1994) Extent of neovascularization as a prognostic indicator in thin (<0.76 mm) melanomas. *Am J Pathol* **145**: 510–514

Greenberg P, Cox C, LeBeau MM, Fenaux P, Morel P, Sanz G, Sanz M, Vallespi T, Hamblin T, Oscier D, Ohyashiki K, Toyama K, Aul C, Mufti G and Bennett J (1997) International scoring system for evaluating prognosis in myelodysplastic syndromes. *Blood* **89**: 2079–2088

Harris AL (1997) Antiangiogenesis for cancer therapy. *Lancet* **349**: 13–15

Lewis CE, Leek R, Harris A and McGee JO (1995) Cytokine regulation of angiogenesis in breast cancer: the role of tumor-associated macrophages. *J Leuk Biol* **57**: 747–751

Linderholm B, Tavelin B, Grankvist K and Henriksson R (1998) Vascular endothelial growth factor is of high prognostic value in node-negative breast carcinoma. *J Clin Oncol* **16**: 3121–3128

Mohle R, Green D, Moore MA, Nachman RL and Rafii S (1997) Constitutive production and thrombin-induced release of vascular endothelial growth factor by human megakaryocytes and platelets. *Proc Natl Acad Sci USA* **94**: 663–668

Nguyen M, Watanabe H, Budson AE, Richie JP, Hayes DF and Folkman J (1994) Elevated levels of an angiogenic peptide, basic fibroblast growth factor, in the urine of patients with a wide spectrum of cancers. *J Natl Cancer Inst* **86**: 356–361

Perez-Atayde AR, Sallan SE, Tedrow U, Connors S, Allred E and Folkman J (1997) Spectrum of tumor angiogenesis in the bone marrow of children with acute lymphoblastic leukemia. *Am J Pathol* **150**: 815–821

Salven P, Teerenhovi L and Joensuu H (1997) A high pretreatment serum vascular endothelial growth factor concentration is associated with poor outcome in non-Hodgkin's lymphoma. *Blood* **90**: 3167–3172

Solanilla A, Grosset C, Lemercier C, Dupouy M, Mahon FX, Schweitzer K, Reiffers J, Weksler B and Ripoche J (1998) Expression of Flt3-ligand by the endothelial cell. Regulation by IL-1$\alpha$, glucocorticoids, IFN-$\alpha$, MIP-1$\alpha$ and TGF$\beta$. Key role in the proliferation of primitive hematopoietic progenitors. *Blood* **92**: 580a (abstract)

Vacca A, Ribatti D, Roncali L, Ranieri G, Serio G, Silvestris F and Dammacco F (1994) Bone marrow angiogenesis and progression in multiple myeloma. *Br J Haematol* **87**: 503–508

Weidner N, Semple JP, Welch WR and Folkman J (1991) Tumor angiogenesis and metastasis – correlation with invasive breast carcinoma. *N Engl J Med* **324**: 1–8

Yamaguchi H, Ishii E, Saito S, Tashiro K, Fujita I, Yoshiodomi S, Ohtubo M, Akazawa K and Miyazaki S (1996) Umbilical vein endothelial cells are an important source of c-kit and stem cell factor which regulate the proliferation of haemopoietic progenitor cells. *Br J Haematol* **94**: 606–611

© 1999 Cancer Research Campaign
